# Supplementary material for: Large attachment organelle mediates interaction between Nanobdellota archaeon YN1 and its host
Source: ISME J. 2024 Aug 8;18(1):wrae154. doi: 10.1093/ismejo/wrae154 (PMC11420986; doi:10.1093/ismejo/wrae154)
Supplement: SI_26072024_CleanVersion_wrae154 [file si_26072024_cleanversion_wrae154.docx]

# Supplementary Information for

# Large attachment organelle mediates interaction between a novel Nanobdellota archaeon YN1 and its host

Matthew D Johnson^1,2,#^, Hiroyuki D. Sakai^3,4, #^, Bindusmita Paul^1,2,†^, Takuro Nunorura^5,†^, Somavally Dalvi^1,2,†^, Manasi Mudaliyar^1,2^, Doulin C Shepherd^1,2^, Michiru Shimizu^4^, Shubha Udupa^1,2^, Moriya Ohkuma^4^, Norio Kurosawa^3^, Debnath Ghosal^1,2,*^


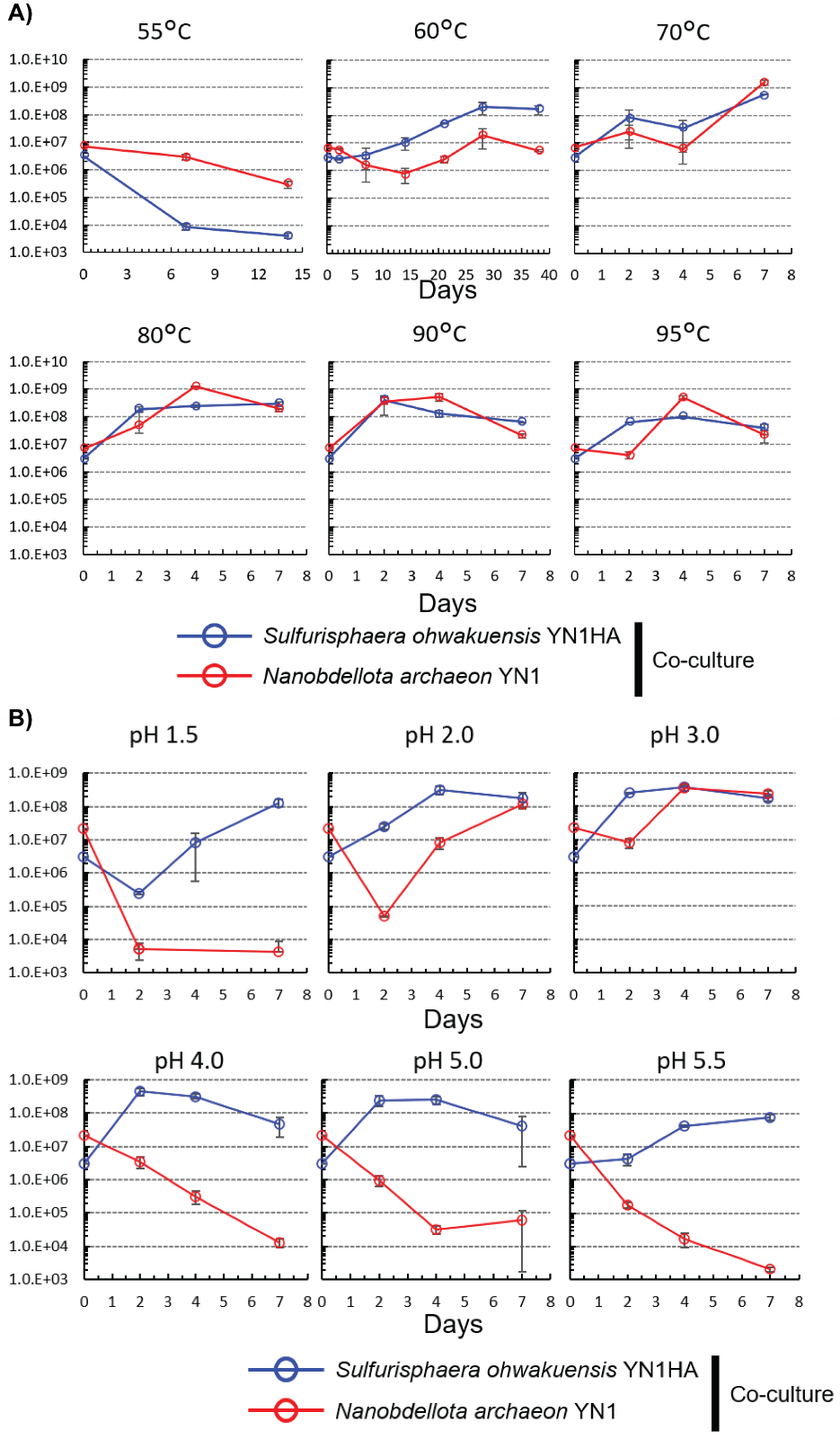


Supplementary figure 1. Optimal growth conditions for the YN1 – YN1HA co-culture system. (A) growth curves of YN1 and YN1HA at different temperatures. (B) Growth curves of YN1 and YN1HA at different pH. The error bars indicate the standard deviation based on two culture replicates.　　　
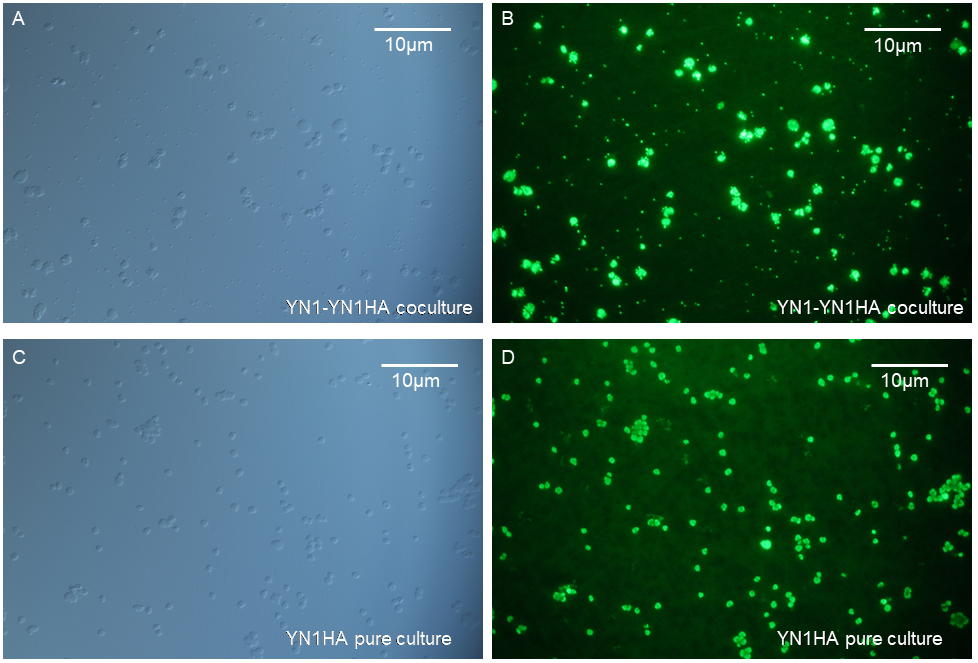


Supplementary figure 2. Differential interference contrast microscopy (A, C) and fluorescence microscopy (B, D) of YN1-YN1HA co-culture (A, B) and YN1HA pure culture (C, D) stained with SYBR green.
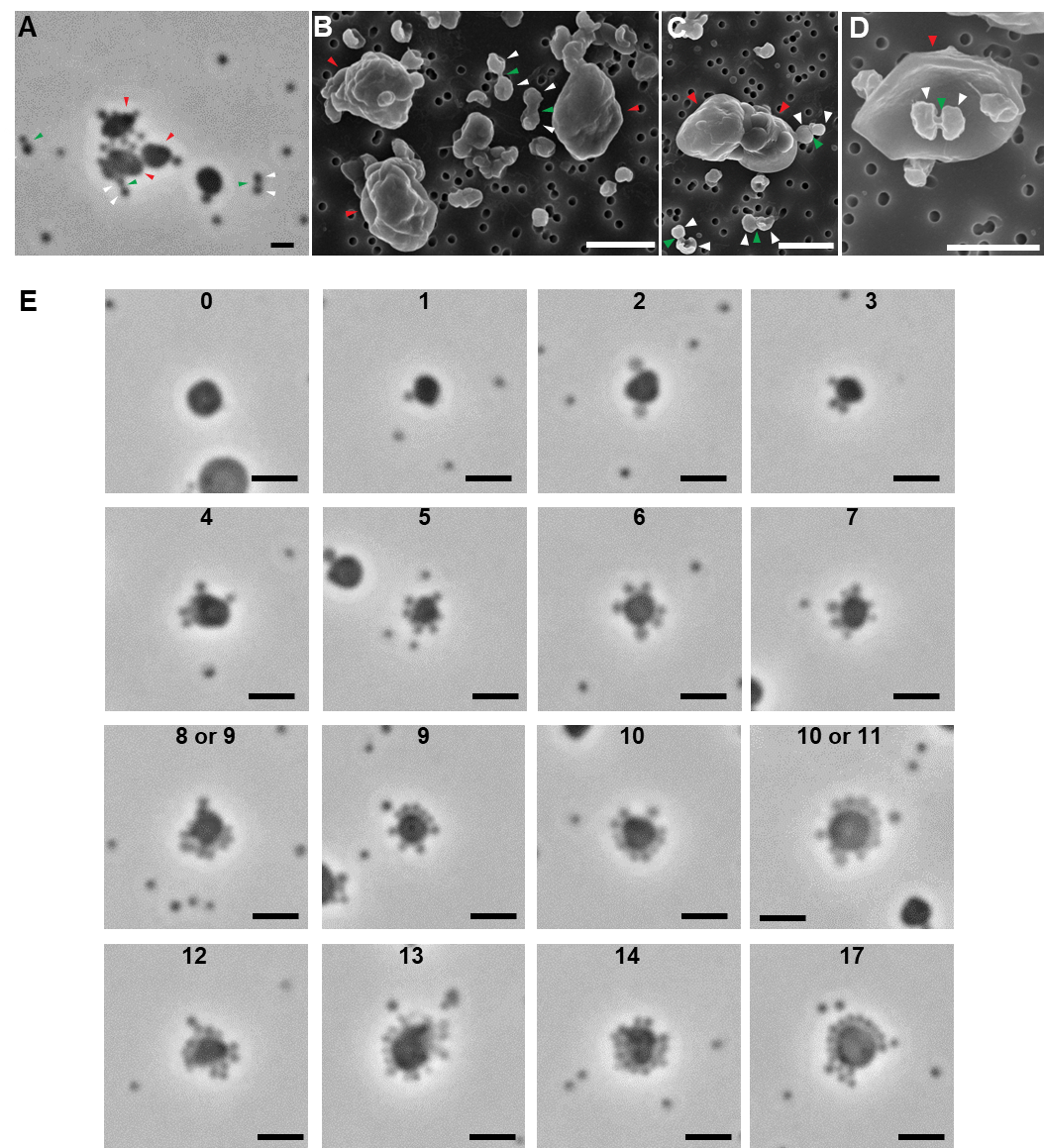


**Supplementary figure 3.** Phase contrast microscopy and SEM analysis showing example cell division events of YN1 cells separate from the host cell (A, B, C) and on the surface of host cell (A, C, D). Red, white, and green arrows indicate YN1HA cells, YN1 cells, and YN1 cell division sites respectively. (E) Phase contrast microscopy of YN1-YN1HA coculture at day 4 showing the number of YN1 cells attaching to the cell surface of a YN1HA cell. The numbers on each image indicate the number of YN1 cells attached to the YN1HA cell. Scale bars represent 2 µm.


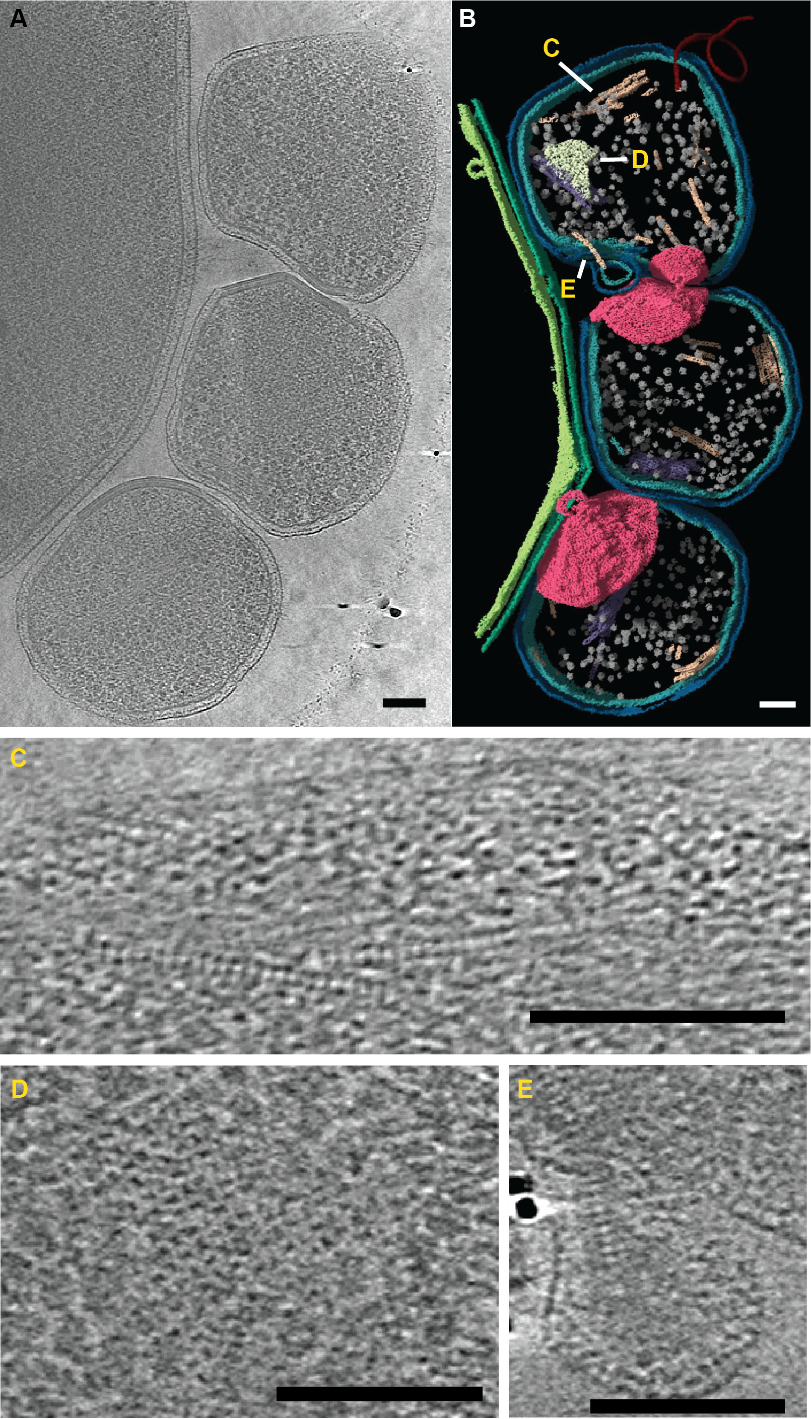


Supplementary figure 4. Additional features in YN1 cells. (A) 2-D slice through a 3-D tomogram showing a host interacting with three YN1 cells. (B) Segmented volume of the same tomogram as in (A), highlighting interesting features. (C-E) 2-D slices from the same tomogram shown in (A) focussing on the interesting features, (C) striated filaments, (D) 2D matrix, (E) filaments inside a membrane protrusion. Scale bars represent 100 nm.

## Supplementary Information Movie legends:

### SI Movie 1

Phase contrast microscopy at room temperature of the YN1 DPANN interacting with its host YN1HA. DPANN cells appear to be stationary on host cells while the host oscillates.

### SI Movie 2

Tomographic movie moving through the z axis showing the interaction of YN1 DPANN cells to their YN1HA host cell. Also shown are the intercellular and cytoplasmic filaments.

### SI Movie 3

Three-dimensional (segmented) view of an YN1 and YN1HA interaction showing examples of the attachment organelles connecting the two cell types. Also shown are the intercellular and cytoplasmic filaments.

### SI Movie 4

Tomographic movie moving through the z axis showing the interaction of YN1 cells forming various interactions with a YN1HA host cell. Also shown are the intercellular, cytoplasmic, and sheath filaments

### SI Movie 5

Three-dimensional (segmented) view of a YN1 cells forming various interactions with a YN1HA host cell. Also shown are the intercellular, cytoplasmic, and sheath filaments.

### SI Movie 6

Tomographic movie through the z axis of a YN1 cell searching for a host, showing the presence the attachment organelle before host cell interaction.

### SI Movie 7

Tomographic movie moving through the z axis showing two YN1 cells in proximity with their attachment organelles forming the interacting surfaces.

## Supplementary Tables

### Supplementary table 1

YN1 genome annotation.

### Supplementary table 2

YN1HA genome annotation.

### Supplementary table 3

Quantification of features observed in cryotomograms.
